# Knowledge and Awareness of Adults towards Nocturnal Enuresis in Children among the Medina Population

**DOI:** 10.3390/children11060640

**Published:** 2024-05-25

**Authors:** Muayad Saud Albadrani, Tariq Abdulazez Alluqmani, Osama Hamed Alsehli, Meshal Saleem Alsaedi, Hassan Saleh Alrehaili, Ahmed Abdullah Aljabri, Muhammad Abubaker Tobaiqi

**Affiliations:** 1 Department of Family and Community Medicine and Medical Education, College of Medicine, Taibah University, Al-Madinah 42353, Saudi Arabia; 2 College of Medicine, Taibah University, Al-Madinah 42353, Saudi Arabia; tu4002106@taibahu.edu.sa (T.A.A.); tu4003166@taibahu.edu.sa (O.H.A.); tu4003163@taibahu.edu.sa (M.S.A.); tu4000395@taibahu.edu.sa (H.S.A.);

**Keywords:** nocturnal enuresis, knowledge, awareness, adults, children, medina, Saudi Arabia

## Abstract

**Background:** Nocturnal Enuresis (NE) is a common problem among children that is stressful for both the child and adults. There is a lack of adults’ knowledge and awareness of the NE condition. **Objective:** This study aimed to evaluate the adults’ knowledge and awareness of NE in Medina City, Saudi Arabia. **Method:** A cross-sectional observational study was conducted among adults in Medina through September and October 2023, using a questionnaire composed of socio-demographic characteristics and adults’ knowledge and awareness of NE. A statistical analysis was performed using SPSS software. **Results:** The study was conducted among 553 adults in Medina, with a mean (standard deviation [SD]) age of 37.69 (10.775). Most participants (94.8%) were Saudi nationals, of which 84.4% were females, 76.3% were married, and 97.1% were urban residents with university degrees (80.3%). The mean (SD) total score of knowledge and awareness was 4.69 (1.783) out of 9 and 6.49 (2.167) out of 12, respectively. Being female (*p* < 0.001), with a university degree (*p* = 0.002), and knowing about enuresis in children (*p* = 0.011) are significant factors affecting adults’ knowledge with higher scores than others. **Conclusions:** An inadequate knowledge and awareness level of NE in children was revealed among adults living in Medina City, Saudi Arabia. These results emphasize the need for targeted educational campaigns to enhance adults’ knowledge and awareness of enuresis.

## 1. Introduction

Nocturnal enuresis (NE) is a condition where a child aged five or older involuntarily urinates during sleep. As per the Diagnostic and Statistical Manual of Mental Disorders (DSM V), NE is diagnosed if a child wets their bed or clothes at least twice a week for three consecutive months, causing significant distress or impairment in multiple areas such as intellectual and social functioning [1]. NE is categorized into primary and secondary types. Primary NE, the more prevalent form, accounts for 90% of cases and is typically linked to a family history of NE. It may also result from developmental issues affecting the urinary bladder sphincter. Secondary NE is often due to underlying conditions like recurrent urinary tract infections (UTIs), spinal cord disorders, diabetes mellitus (DM), or psychological stress [2]. Several risk factors are attributed to NE development, such as family history, poor parental education, low birth weight, and psychological stress [3]. It has been proven that Nocturnal enuresis is associated with hereditary factors. Common genetic variants contribute considerably to NE, identifying potential NE risk genes with roles in sleep, urine production, and bladder function [4]. An underactive bladder may lead to incomplete voiding and elevated abdominal pressure during voiding, resulting in an interrupted uroflow curve [5].

NE is the second most common illness affecting children aged 6 to 14 years old, after allergic conditions. It is a frequent problem among school-aged children and can cause issues in many aspects of the child’s life, as well as their parents. These issues may include emotional or behavioral problems, which can be more harmful to the child than the enuresis itself. The prevalence rate of NE varies between 5% to 20% worldwide and 15% in Saudi Arabia [1,2].

The clinical presentation further divides NE into two categories: MNE (monosymptomatic nocturnal enuresis) and non-MNE. MNE is used for NE alone, while non-MNE is used with additional symptoms present [5]. The diagnosis of NE requires a thorough history from the patient and parents, as well as a clinical examination to rule out secondary NE [6]. There are two primary ways to treat a patient with NE. One approach is a behavioral strategy that involves the use of alarms, rewards, and a reduction in fluid consumption at night. The second approach is to use medications such as oxybutynin, imipramine, and desmopressin. Bed alarms are considered to be the first-line therapy as they help reduce the recurrence rate of NE [6,7].

In the northern region of Saudi Arabia, it was found that 76% of mothers noticed an improvement in their children’s condition by reducing their fluid intake. Additionally, 66% of mothers woke their children up to go to the bathroom. The study also revealed that 71% of moms feel ashamed and embarrassed. Moreover, the study showed a strong relationship between NE and increased stress levels [8]. 

Many caregivers assume that NE will stop as children grow older. This is why some delay seeking medical treatment for NE until it becomes a significant issue for both the child and the adults [9]. In order to reduce the devastating consequences of NE caused by adults’ lack of knowledge, this study aimed to evaluate the adults’ knowledge and awareness of NE in Medina City.

## 2. Methodology

### 2.1. Study Design

This observational cross-sectional study was performed among adults in Medina, Saudi Arabia. The data collection was through September and October 2023. 

### 2.2. Study Population

The study included adults aged 18 to 65 who lived in Medina City within the catchment area and were willing to participate. Participants who were less than 18 years old were excluded from the study.

### 2.3. Sample Size Calculation

Based on a 95% confidence interval, a 5% margin of error, and a 0.05 significance level, a minimum of 384 participants should be included in this study. 

### 2.4. Data Collection

Data were collected through a structured and self-administered electronic questionnaire randomly via social media using Google Forms. A pilot study was conducted for our structured questionnaire. The questionnaire was initially developed in English, and then the researchers translated the questionnaire into Arabic to be understood by the target audience. The data collection tool consisted of three sections. The first section included the participants’ characteristics such as age, gender, residency, educational level, social status, and number of children. The second section included questions about the adults’ knowledge of NE. These questions included causes, types of NE, risk factors, and the different methods of management. The third section included questions about the parent’s awareness of their children’s social and psychological assessment.

### 2.5. Data Entry and Analysis

The data were extracted and reviewed using Excel. Statistical analyses (Build 16327.20214 2304: version Number Microsoft 365 Apps for Enterprise: Editio:) were performed with IBM SPSS software (version 26.0, Armonk, NY, USA). Categorical variables were presented as frequencies and percentages. The Chi-square test and Fisher’s Exact Test were employed to compare categorical variables, with statistical significance defined as a *p*-value less than 0.05.

## 3. Results

The study included 553 participants aged 18 to 62 years, with a mean (standard deviation [SD]) age of 37.69 (10.775). Most participants (94.8%) were Saudi nationals, of which 84.4% were females, 76.3% were married, and 97.1% were urban residents with university degrees (80.3%). Additionally, 75.9% of the participants had children, and 73.6% had 1–5 children, as shown in Table 1.

Table 2 illustrates the frequency and percentage distribution of NE.

Regarding knowledge, 73.1% of the participants thought that enuresis is abnormal. The majority of the participants responded that psychological and social factors such as family breakdown or child abuse (68.2%) were the causes of childhood enuresis. Furthermore, organic causes such as urinary system infections, a small bladder, diabetes, severe abdominal pain (53%), frequent fluid consumption (56.15%), hereditary influences (24.2%), and deep sleep (27.7%) were also reported as common causes. About half of the participants (48.6%) said that enuresis could be diagnosed in a more than five-year-old child. Moreover, 66% of the participants stated that a child should urinate a certain number of times in the middle of the night to be diagnosed with enuresis. Additionally, 79% of them reported that the child should urinate more than twice a month to be diagnosed with enuresis.

In terms of awareness, the majority of participants revealed that their motivation for seeking treatment was the worry that enuresis could negatively impact their child’s interpersonal relationships (67.5). A smaller percentage (23.5%) worried that sleep disturbance due to enuresis may cause growth retardation. Almost all participants (94.4%) acknowledged that there are effective treatments available for enuresis. Additionally, most participants (73%) believed that medical and behavioral solutions exist. Only 6.5% of the participants thought setting up a timer was the most effective way to manage NE. Furthermore, a significant number of participants (90.6%) believed that enuresis can have a negative impact on a child’s psychological well-being. Specifically, participants reported that a lack of self-confidence (69.7%), shyness and isolation (77.2%), and even aggressiveness (15.6%) were psychological effects of NE. The majority of participants (72.9%) believed that children with enuresis should see a doctor if they still experience urine incontinence after the age of five. Additionally, 37.8% reported the presence of urinary tract infection or daytime urine incontinence symptoms, and 37.1% reported a loss of the ability to withhold urine after a period of normal urination as reasons to consult a doctor.

The total scores of the attitude and perception regarding NE among the participants are described in Table 3. The total knowledge score ranged from 1 to 9 out of 9, with a mean (SD) of 4.69 (1.783). Moreover, the total score of the awareness ranged from 1 to 12 out of 12, with a mean (SD) of 6.49 (2.167).

Regarding knowledge, there was a significant correlation between gender (*p* < 0.001), educational level (*p* = 0.002), number of children (*p* = 0.001), and if the participants know anything about enuresis in children (*p* = 0.011) with the knowledge score NE. Females had a higher knowledge score than males, with a mean (SD) of 4.80 (1.747) vs. 4.06 (1.856), respectively. The participants with university degrees had a higher knowledge score than those with less than a university degree, with a mean (SD) of 4.81 (1.799) vs. 4.19 (1.636), respectively. The participants who had 1–3 children, with a mean (SD) of 4.91 (1.862), had the highest knowledge score than those who had more than three and no children, with a mean (SD) of 4.61 (1.688) and 4.03 (1.622), respectively. The participants who reported that they knew enuresis in children had a higher knowledge score than those who reported that they did not have, with a mean (SD) of 4.78 (1.792) vs. 4.33 (1.711), respectively. However, there was no significant correlation between the knowledge scores and age, nationality, marital status, residency, and having children.

Regarding awareness, there was a significant correlation between age (*p* = 0.037), gender (*p* = 0.019), educational level (*p* < 0.001), and if the participants know anything about enuresis in children (*p* = 0.007) with the awareness score NE. The participants aged less than or equal to 35 years had a higher awareness score than those aged more than 35 years, with a mean (SD) of 6.69 (2.297) vs. 6.33 (2.052), respectively. Females had a higher awareness score than males, with a mean (SD) of 6.58 (2.170) vs. 5.95 (2.086), respectively. The participants with university degrees had a higher awareness score than those with less than a university degree, with a mean (SD) of 6.71 (2.101) vs. 5.59 (2.208), respectively. The participants who reported that they had knowledge about enuresis in children had a higher awareness score than those who reported that they did not have, with a mean (SD) of 6.61 (2.098) vs. 6.01 (2.369), respectively. However, there was no significant correlation between the awareness scores and nationality, marital status, residency, having children, and number of children, as shown in Table 4.

## 4. Discussion

This study evaluated adults’ knowledge and awareness of NE in the Madina population. The findings were promising, revealing moderate knowledge and awareness among adults in Medina. In addition, females with a university degree and prior knowledge about enuresis showed greater knowledge and awareness than the other participants. 

Our results were inconsistent with a study in Egypt that revealed a good level of knowledge and attitude regarding enuresis in general [9]. However, in Taif, there was a lack of knowledge regarding the presence of NE; even the majority of participants believed that NE is caused by several factors but were unaware of its different types [8]. Another study showed a lack of general parental knowledge of the causes and effective treatments for NE. About half of them reported they would seek medical care for their child with NE, and one-third reported awareness of effective treatments [7]. Moreover, Magura R. found that there was a lack of knowledge and awareness among parents [10]. In Tabuk, Saudi Arabia, two-thirds of the participants had no knowledge of the causes of NE, while the majority were aware of NE as a medical problem [11]. 

Similar to our results, a study in Taif City and Tabuk found that individuals with higher educational qualifications had significantly more prior knowledge about enuresis than those with lower educational qualifications [9,11]. Females and those with graduate-level education were more likely to seek medical care for their child with NE [7]. It was also proved that parental education level was related to the management of enuresis [12].

The majority of the participants believed that the main reason for seeking treatment for NE was the negative psychological impact it had on the children. This indicates a good level of awareness since it is widely known that NE can have adverse psychological effects on children. Previous studies have shown that NE can lead to lower self-image, self-esteem, and social withdrawal. The impact of NE tends to be more severe with an increase in age [13,14]. 

Our research highlights the importance of improving knowledge and awareness of NE, the need for a deeper understanding of its treatment effectiveness, and the elimination of misconceptions. To achieve this goal, various methods can be employed, including campaigns, social media, school health programs, and discussions during health interviews between healthcare providers and adults, especially parents.

## 5. Limitation

The study’s findings are constrained by its cross-sectional observational design, which utilized an online questionnaire conducted in a single region of Saudi Arabia, capturing data at one point in time. This approach may not account for temporal variations. Additionally, not all potential factors influencing knowledge and awareness of NE were considered. Future research should employ more generalized study designs and comprehensive investigations to encompass all variables that could affect adults’ knowledge and awareness of NE (See Appendix A).

## 6. Conclusions

The study found that adults in Medina City, Saudi Arabia, have a moderate level of knowledge and awareness regarding NE in children. Hence, it is important to implement educational programs aimed at enhancing awareness and understanding of enuresis through community workshops, seminars, parenting classes, and online platforms. Broad-reaching awareness campaigns are necessary to improve public comprehension of enuresis. Moreover, collaboration between researchers, healthcare providers, and educational institutions is very important to ensure that accurate information is disseminated to adults, particularly parents.

## Figures and Tables

**Table 1:** Demographic characteristics of the participants (N = 553).

**Age (Years) (546)**	Mean (SD)	37.69 (10.775)	
	Median (IQR)	38 (16)	
	Min–Max	18–62	
**Parameters**	**Category**	**N**	**Percentage**
**Gender**	Male	86	15.6
	Female	467	84.4
**Nationality**	Saudi	524	94.8
	Non-Saudi	29	5.2
**Educational level**	Illiterate	5	0.9
	Secondary school	30	5.4
	High school	74	13.4
	University	444	80.3
**Marital status**	Single	93	16.8
	Married	422	76.3
	Divorced	29	5.2
	Widowed	9	1.6
**Residency**	Rural	16	2.9
	Urban	537	97.1
**Do you have children?**	No	133	24.1
	Yes	420	75.9
**Number of children (N = 551)**	Zero	61	11.1
	One	70	12.7
	Two	100	18.1
	Three	91	16.5
	Four	82	14.9
	Five	63	11.4
	Six	34	6.2
	Seven	22	4.0
	Eight	18	3.3
	Nine	6	1.1
	Ten	2	0.4
	Twelve	2	0.4
**Do you already know anything about enuresis in children?**	No	149	26.9
	Yes	404	73.1

SD: Standard Deviation. IQR: Interquartile range. N: Number.

**Table 2:** Frequency and percentage distribution regarding NE (N = 553).

Questions		N	Percentage
Knowledge			
Do you think that enuresis is abnormal?	No	149	26.9
	Yes	404	73.1
What, in your opinion, are the causes of childhood enuresis?	Organic causes (infections of the urinary system, a small bladder, diabetes, or severe abdominal pain)	293	53.0
	Psychological and social, such as family breakdown or abuse towards children	377	68.2
	Hereditary influences	134	24.2
	Frequent fluid consumption	310	56.1
	Deep sleep	153	27.7
What age may enuresis be diagnosed in a child?	3–4 years	145	26.2
	Five years	139	25.1
	More than five years	269	48.6
Does a patient have to urinate in the middle of the night a particular number of times to be diagnosed with enuresis?	No	188	34.0
	Yes	365	66.0
If the answer to the previous question is yes, how many times should the child urinate to be diagnosed with enuresis (N = 362)	Once a month	28	7.7
	Twice a month	48	13.3
	More than twice a month	286	79
**Awareness**			
What are the motives for treatment? (N = 548)	Worry about that the child’s enuresis condition may get worse.	269	49.1
	Worry that the child’s enuresis might result in another urinary health issue.	274	50
	Worrying about that enuresis condition may harm a child’s social interactions.	370	67.5
	Worry that disruptions in sleep due to enuresis could delay growth.	129	23.5
Do you have any effective methods to treat enuresis? (N = 551)	No	31	5.6
	Yes	520	94.4
If the previous question was answered yes, what type of solutions exist? (N = 519)	Medical treatment	37	7.1
	Behavioral treatment	71	13.7
	Both medical and behavioral	379	73
	Other types of treatment	31	6
What psychological treatments do you think are most effective for treating nocturnal enuresis?	Setting up a timer	36	6.5
	Educate kids on bladder control for increased bladder volume	75	13.6
	Limiting fluids before sleeping time	208	37.6
	Mental support	189	34.2
	I don’t know	45	8.1
Do you believe that enuresis impacts a child’s psychological well-being?	Yes	501	90.6
	No	19	3.4
	I don’t know	33	6
If the previous question was answered yes, what psychological effects does NE have? (N = 501)	Lacking in self-confidence	349	69.7
	Shyness and isolation	387	77.2
	Aggressiveness	78	15.6
	I don’t know	29	5.8
When should someone with enuresis, in your opinion, see a doctor?	Still having urine incontinence after the age of five	403	72.9
	The presence of urinary tract infection or if there are symptoms of urine incontinence during the day	209	37.8
	After a period of normal urination, the ability to withhold urine is lost	205	37.1
	At any age and whenever the child urinates in bed	139	25.1
	I don’t know	46	8.3

**Table 3:** Total scores of the participants’ answers regarding attitude and perception towards NE.

**The total score of the knowledge of NE**	**Mean (SD)**	4.69 (1.783)
	**Median (IQR)**	5 (3)
	**Min–Max**	1–9
**The total score of the awareness of NE**	**Mean (SD)**	6.49 (2.167)
	**Median (IQR)**	6 (3)
	**Min–Max**	1–12

**Table 4:** Correlation between the individuals’ characteristics and attitude and perception scores regarding NE.

Factors		Mean (SD)	Median (IQR)	*p*-Value
Knowledge				
Age	≤35 years	4.82 (1.901)	5 (3)	0.139
	>35 years	4.58 (1.683)	4 (3)	
Gender	Male	4.06 (1.856)	4 (2)	**<0.001**
	Female	4.80 (1.747)	5 (2)	
Nationality	Saudi	4.70 (1.785)	5 (3)	0.438
	Non-Saudi	4.45 (1.764)	4 (3)	
Educational level	Less than a university degree	4.19 (1.636)	4 (2)	**0.002**
	University degree	4.81 (1.799)	5 (2)	
Marital status	Married	4.71 (1.743)	5 (3)	0.463
	Unmarried	4.61 (1.912)	4 (3)	
Residency	Rural	4.56 (1.861)	4.5 (3)	0.675
	Urban	4.69 (1.783)	5 (3)	
Do you have children?	Yes	4.75 (1.769)	5 (3)	0.106
	No	4.48 (1.820)	4 (3)	
Number of children	No children	4.03 (1.622)	4 (2)	**0.001**
	1–3	4.91 (1.862)	5 (2)	
	More than 3	4.61 (1.688)	5 (3)	
Do you already know anything about enuresis in children	Yes	4.78 (1.792)	5 (3)	**0.011**
	No	4.33 (1.711)	4 (2)	
**Awareness**				
Age	≤35 years	6.69 (2.297)	7 (3)	**0.037**
	>35 years	6.33 (2.052)	6 (3)	
Gender	Male	5.95 (2.086)	6 (4)	**0.019**
	Female	6.58 (2.170)	6 (3)	
Nationality	Saudi	6.51 (2.163)	6 (3)	0.348
	Non-Saudi	6.14 (2.248)	6 (2)	
Educational level	Less than a university degree	5.59 (2.208)	5 (3)	**<0.001**
	University degree	6.71 (2.101)	7 (3)	
Marital status	Married	6.42 (2.042)	6 (3)	0.124
	Unmarried	6.69 (2.526)	7 (4)	
Residency	Rural	6.94 (2.435)	6.5 (4)	0.500
	Urban	6.47 (3)	6 (3)	
Do you have children?	Yes	6.47 (2.063)	6 (3)	0.512
	No	6.54 (2.476)	7 (4)	
Number of children	No children	5.98 (2.540)	6 (4)	0.183
	1–3	6.61 (2.226)	7 (3)	
	More than 3	6.48 (1.976)	6 (3)	
Do you already know anything about enuresis in children	Yes	6.61 (2.098)	7 (3)	**0.007**
	No	6.01 (2.369)	6 (4)	

## Data Availability

Data are available from the corresponding author upon reasonable request due to (specify the reason for the restriction: e.g., privacy, legal or ethical reasons).

## Appendix A. Questionnaire

**Question**	**Answer**
(1) Gender	- Male - Female
(2) Age	
(3) Nationality	- Saudi - Non-Saudi
(4) Educational level	- University - High School - Secondary School - Illiterate
(5) Marital status	- Married - Divorced - Widowed - Single
(6) Residency	- Urban - Rural
(7) Do you have children?	- Yes - No
(8) Number of children in the family:	
Knowledge	
(9) Do you already know anything about enuresis in children?	- Yes - No
(10) Do you think that enuresis is abnormal?	- Yes - No
(11) What, in your opinion, are the causes of childhood enuresis?	- Organic causes (Infections of the urinary system, a small bladder, diabetes, or severe abdominal pain) - Psychological and social, such as family breakdown or abuse towards children - hereditary influences - frequent fluid consumption - deep sleep
(12) What age may enuresis be diagnosed in a child?	- 3–4 years - 5 years - More than 5 years
(13) Does a patient have to urinate in the middle of the night a particular number of times to be diagnosed with enuresis?	- Yes - No
(14) If the answer to the previous question is yes, how many times should the child urinate to be diagnosed with enuresis	- once a month - Twice a month - More than twice a month
Awareness	
(15) What are the motives for treatment?	- Worry that the child’s enuresis will get worse. - Worry that the child’s enuresis will develop into another urologic disease. - Worry that enuresis negatively affects the interpersonal relationships of my child. - Worry that sleep disturbance because of enuresis may induce growth retardation.
(16) Are there any effective methods to treat enuresis, in your opinion?	- Yes - No
(17) If the previous question was answered yes, what solutions exist?	- Medical treatment - Behavioral treatment - Both medical and behavioral - Other types of treatment
(18) What psychological treatments do you think are most effective for treating nocturnal enuresis?	- Setting up a timer - Educate kids on bladder control for increased bladder volume - Limiting fluids before sleeping time - Mental support - I don’t know
(19) Do you believe that enuresis has an impact on a child’s psychological well-being?	- Yes - No - I don’t know
(20) If the previous question was answered yes, what psychological effects does nocturnal enuresis have?	- Lacking in self-confidence - Shyness and isolation - Aggressiveness - I don’t know
(21) When should someone with enuresis, in your opinion, see a doctor?	- Still having urine incontinence after the age of five - The presence of urinary tract infection or if there are symptoms of urine incontinence during the day - After a period of normal urination, the ability to withhold urine is lost - At any age and whenever the child urinates in bed - I don’t know

## References

[1] Alhifthy E.H., Habib L., Al-Makarem A.A., AlGhamdi M., Alsultan D., Aldhamer F., Buhlagah R., Almubarak F.M., Almufadhi E., Bukhamsin G.M. (2020). Prevalence of nocturnal enuresis among Saudi children population. Cureus.

[2] Sherah K., Elsharief M., Barkat N., Jafery A. (2019). Prevalence of Nocturnal Enuresis in school-age children in Saudi Arabia. IJMDC.

[3] Goweda R., Badr H., Benjabi W., Kalantan R., Kalantan R. (2020). Nocturnal enuresis among children: Prevalence and risk factors. Med. Sci..

[4] Jørgensen C.S., Horsdal H.T., Rajagopal V.M., Grove J., Als T.D., Kamperis K., Nyegaard M., Walters G.B., Eðvarðsson V.Ö., Stefánsson H. (2021). Identification of genetic loci associated with nocturnal enuresis: A genome-wide association study. Lancet. Child. Adolesc..

[5] Austin P.F., Bauer S.B., Bower W., Chase J., Franco I., Hoebeke P., Rittig S., Walle J.V., von Gontard A., Wright A. (2014). The standardization of terminology of lower urinary tract function in children and adolescents: Update report from the Standardization Committee of the International Children’s Continence Society. J. Urol..

[6] Tai T.T., Tai B.T., Chang Y.-J., Huang K.-H. (2021). The Importance of Understanding Parental Perception When Treating Primary Nocturnal Enuresis: A Topic Review and an Institutional Experience. Res. Rep. Urol..

[7] Schlomer B., Rodriguez E., Weiss D., Copp H. (2013). Parental beliefs about nocturnal enuresis causes, treatments, and the need to seek professional medical care. J. Pediatr. Urol..

[8] Al-Jaid M.S., Alotaibi N.M., Alrbeeai H.A. (2019). Knowledge and Awareness of Community about Pediatric Nocturnal Enuresis in Taif City, 2019. Fam. Med..

[9] Mohamed Abd-Elrazik Khalil N., Abu Bakr O., EzzEldin Prince H. (2021). Awareness of Parents Having School Age Children with Enuresis. Egypt. J. Health Care.

[10] Magura R. (2015). Nocturnal enuresis in children. Pharm. J..

[11] Alomani O.T., Khalil T. (2021). Nocturnal Enuresis in Primary Schools Children (6–12 Years) of Tabuk City, Saudi Arabia. J. Pharm. Res. Int..

[12] Pillai R.R., Sara B. (2019). Parental attitudes and perceptions regarding primary nocturnal enuresis in children. Int. J. Curr. Res..

[13] Theunis M., Van Hoecke E., Paesbrugge S., Hoebeke P., Vande Walle J. (2002). Self-image and performance in children with nocturnal enuresis. Eur. Urol..

[14] Theunis M., Van Hoecke E., Paesbrugge S., Hoebeke P., Walle J. (2014). Impact of enuresis nocturna on health-related quality of life in children and their mothers. J. Pediatr. Urol..

